# From Craft to Reflective Art and Science: Comment on "Metrics and Evaluation Tools for Patient Engagement in Healthcare Organization- and System-Level Decision-Making: A Systematic Review"

**DOI:** 10.15171/ijhpm.2018.108

**Published:** 2018-11-11

**Authors:** Antoine Boivin

**Affiliations:** Center of Excellence on Partnership with Patients and the Public, University of Montreal Hospital Research Center (CRCHUM), Montreal, QC, Canada.

**Keywords:** Patient and Citizen Engagement, Evaluation, Health Research, Policy

## Abstract

Patient engagement practices are increasingly incorporated in health research, governance, and care. More recently, a large number of evaluation tools and metrics have been developed to support engagement evaluation. This growing interest in evaluation reflects a maturation of the patient engagement field, moving from a "craft" to a reflective "art and science," with more explicit expected benefits and risks, better understood conditions for success and failure, and increasingly rigorous evaluation instruments to improve engagement theories and interventions. It also supports a more critical view of engagement science, moving beyond reductionist views of engagement as a "black box technology" to a more subtle view of this broad category of complex interventions. Structured evaluation can advance patient engagement by supporting more reflective partnerships between patients, clinicians, health system leaders and citizens. This can help clarify mutual (and potentially contradictory) expectations toward engagement, provide a reality check toward claims of benefits and harms, and increase health systems’ capacity to implement effective engagement practices over time. To do so, closer collaborations are required between engagement scientists and practitioners to align the theories, practice and evaluation of patient and community engagement.

## Introduction

Patient engagement practices and programs and increasingly being incorporated in different areas of healthcare, including health policy,^1^ quality improvement,^2^ health technology assessment,^3^ clinical practice guidelines,^4^ research,^5^ priority-setting,^6^ and clinical care.^7^ More recently, a number of evaluation tools and metrics have been developed to support engagement evaluation. Searching the literature from 1973 to 2015, Dukhanin and colleagues identified 23 evaluation tools for patient engagement in healthcare organizations and system-level decision-making, 87% of which were published after 1996.^8^ A similar growth in evaluation instruments for patient engagement in clinical care, research and community health programs was found in other complementary systematic reviews.^9-11^

## Mapping Evaluation Metrics: The Need to Align Theories and Practice

An original contribution of Dukhanin’s review is its proposed taxonomy of evaluation metrics. The authors built this taxonomy inductively, through reviewers’ descriptive understanding of what each metric was intended to measure. This mapping is helpful to illustrate which dimensions of engagement have received more or less measure development efforts (eg, the dominance of process over outcome measures). The taxonomy could have been strengthened by distinguishing engagement structures (eg, resources and training provided to participants and staff) from engagement processes (eg, respect, trust and transparency).^12^ Careful alignment between engagement theories and evaluation metrics is also necessary to properly interpret and use such taxonomy, given the complexities of the engagement field. For example, using process evaluation criteria based on participants’ representativeness may be appropriate for engagement methods that consult with large numbers of patients and community members (eg, focus groups and surveys) but could be inappropriate for partnership methods where a patient selected on the basis of his experiential knowledge and competencies co-leads a project with a clinician.^13^

## The Maturation of the Field

Overall, the growing interest in patient engagement evaluation documented in this systematic review reflects the maturation of the field, moving from a “craft” (with rarely evaluated engagement initiatives) to an “art and science” (with more explicit expected benefits and risks, better understood conditions for success and failure, and increasingly rigorous evaluation instruments to improve engagement theories and interventions). This evolution of patient engagement practice and evaluation has run through different phases over time.

## An Ethical and Political Imperative

Decades ago, a number of authors and organizations have called for patient and citizen engagement, based on ethical, legal and political principles of autonomy, participative democracy and self-determination. Back in 1969, Sherry Arnstein argued that citizen participation was fundamentally about redistribution of power. At the international level, the Alma-Ata declaration (1978) and the Ottawa Charter (1984) affirmed that people have the right to participate individually and collectively in the planning and implementation of healthcare.^14,15^ These ethical and political principles have spurred the growth of formal engagement structures and mechanisms within government and healthcare organizations (eg, advisory committees, citizen juries, public hearings and consultation).

However, structured evaluation of engagement implementation and its effects on health policies, services and care have often lagged behind, fueling criticisms of “tokenistic engagement.” The focus on process evaluation metrics in Dukhanin’s review may be reflective of a persistently dominant view that “good” patient engagement is primarily about following fair and transparent processes. Evaluating effectiveness also raise specific difficulties. In 1981, Rosener listed four challenges for evaluating the effectiveness of public participation: (1) the complex and value-laden nature of the concept; (2) absence of consensus on criteria to judge success and failure; (3) no agreed-upon evaluation methods; (4) the scarcity of reliable measurement tools.^16^

## Testing the “Black Box Technology”

A number of research and systematic reviews in the 1990s and early 2000s have attempted to answer questions about the effectiveness of patient and citizen engagement, using evidence-based medicine paradigms and methods. This assumed that patient engagement is a technology whose effectiveness, benefits and harms can be tested using similar evaluation methods as other health technologies, like drugs of dialysis (Health Affairs metaphorically dubbed patient engagement the “blockbuster drug of the century”).^17^ Early systematic reviews concluded in the absence of evidence for the effectiveness of patient engagement in collective decision-making (eg, policy-making, research, health technology assessment, clinical guidelines or priority-setting) because of the paucity of randomized controlled trials.^18,19^ More importantly, classic effectiveness studies have left engagement scientists and practitioners living in separate worlds: scientists testing “black box technologies” (does it work?) with little insight about the designs and underlying mechanisms of patient engagement interventions (how does it work?), leaving practitioners with little evidence-based recommendations on how to improve practices.^20^ This divide between engagement scientists and practitioners has also opposed proponents of engagement based on ethical principles, with more instrumental views of engagement whose value would be dependent of its proven impacts.^21^

## Moving Toward a Reflective Art and Science of Engagement

Recent growth in innovative evaluation designs and engagement-specific evaluation metrics have supported more comprehensive approaches to evaluation. For example, the use of process evaluation of cluster randomized trials^22^ and realist evaluations of engagement projects^23,24^ have conceptualized patient engagement as complex interventions whose participants pursue different (potentially contradictory goals) and whose effectiveness is highly dependent upon context.^25^ These evaluations have helped to better understand interactions between engagement context, processes and outcomes. This, in turn, supports the emergence of a reflective approach to the art and science of engagement.

Schon argues that competent practitioners “usually know more than they can say” and exhibit a knowledge in practice that is mostly tacit and intuitive.^26^ From a “reflective art” perspective, evaluation can help engagement practitioners reflect on their own practice and better understand key elements of engagement interventions that explain variations in effectiveness. Looking beyond the technological assumptions of engagement as a set of methods and techniques (eg, deliberative polling vs. nominal group technique), in-depth evaluations of engagement interventions can help understand more subtle aspects of the process (eg, understand how group facilitators influence patients’ expression of opinion and influence).^22,27^ In doing so, evaluation moves from causal analysis to a contribution analysis perspective, seeking to understand the contribution of specific elements (eg, patient engagement) within larger partnerships involving multiple people and organizations.^28^

From a “reflective science” perspective, more critical and nuanced approaches toward evaluation can also help raise awareness of the epistemic tensions raised by patient engagement on the activity of science itself (eg, questioning whose knowledge is recognized as valid).^29^ As patient engagement is increasingly influencing core health science activities (eg, research priority setting, questions, funding and publishing), scientists’ personal attitude toward engagement can influence their interpretation of evidence in the area.^30^

## Building a Coalition of Engagement Practitioners and Scientists

Closer collaboration between engagement practitioners and scientists brought together around “evaluation coalitions” pursing different and complementary evaluation goals (eg, formative evaluation to improve local practices vs. strengthening scientific evidence on engagement), can further support this reflective approach toward the art and science of engagement. Aligned with principles of learning health systems,^31^ the Center of Excellence for Patient and Public Partnership (https://ceppp.ca) offers an example of hub bringing together engagement practitioners and scientists. Co-led by a team of patients, clinicians and researchers, the Center’s aims at improving the practice and science of patient and public partnership in health education, research, and care. From a practice standpoint, the Center’s Partnership School supports engagement interventions (eg, training healthcare leaders and patients to co-lead improvement projects together). Building on these real-world experiments, the Center’s Partnership Lab supports the evaluation of partnership interventions to facilitate improvement of practices over time and contribute to engagement research.^32^ This partnership science experience highlights conditions for successful collaboration between practitioners and researchers (trust building, power-sharing, co-governance) that echo those identified in the community-based participatory research litterature.^23^

## Future Steps

In conclusion, the field of patient engagement evaluation has evolved considerably in the past decades. From an ethical and political ideal, the patient engagement field has matured, both from a practice standpoint (with more refined methods and professionalization of activities such as group facilitation) and an evaluation standpoint (with an increasing number of rigorous evaluation tools). In order to fully contribute to the “art and science” of engagement, greater collaboration is required between engagement practitioners and scientists, while keeping in mind the ethical, epistemological and political tensions that are inherent to patient engagement. More informed dialogue between engagement practitioners and scientists could help clarify mutual (and potentially contradictory) expectations toward engagement, provide a reality check toward claims of benefits and harms, and increase health systems’ capacity to implement effective engagement practices over time.

## Acknowledgements

The author acknowledges the contribution of Audrey l’Esperance, Agustina Gancia, and Alexandre Berkesse from the Center of Excellence on Patient and Public Partnership, Montreal, QC, Canada for comments on previous versions of the manuscript, as well as input from anonymous reviewers.

## Ethical issues

Not applicable.

## Competing interests

Author declares that he has no competing interests.

## Author’s contribution

AB the single author of the paper.

## Funding

AB receives financial support from the Canada Research Chair program (http://www.chairs-chaires.gc.ca).
